# Emergence of an Action Repository as Part of a Biologically Inspired Model of Speech Processing: The Role of Somatosensory Information in Learning Phonetic-Phonological Sound Features

**DOI:** 10.3389/fpsyg.2019.01462

**Published:** 2019-07-10

**Authors:** Bernd J. Kröger, Tanya Bafna, Mengxue Cao

**Affiliations:** ^1^Neurophonetics Group, Department of Phoniatrics, Pedaudiology, and Communication Disorders, Medical School, RWTH Aachen University, Aachen, Germany; ^2^Medical School, RWTH Aachen University, Aachen, Germany; ^3^School of Chinese Language and Literature, Beijing Normal University, Beijing, China

**Keywords:** neural model simulation, speech production and acquisition, speech perception, neural self-organization, connectionism and neural nets

## Abstract

A comprehensive model of speech processing and speech learning has been established. The model comprises a mental lexicon, an action repository and an articulatory-acoustic module for executing motor plans and generating auditory and somatosensory feedback information (Kröger and Cao, 2015). In this study a “model language” based on three auditory and motor realizations of 70 monosyllabic words has been trained in order to simulate early phases of speech acquisition (babbling and imitation). We were able to show that (i) the emergence of phonetic-phonological features results from an increasing degree of ordering of syllable representations within the action repository and that (ii) this ordering or arrangement of syllables is mainly shaped by auditory information. Somatosensory information helps to increase the speed of learning. Especially consonantal features like place of articulation are learned earlier if auditory information is accompanied by somatosensory information. It can be concluded that somatosensory information as it is generated already during the babbling and the imitation phase of speech acquisition is very helpful especially for learning features like place of articulation. After learning is completed acoustic information together with semantic information is sufficient for determining the phonetic-phonological information from the speech signal. Moreover it is possible to learn phonetic-phonological features like place of articulation from auditory and semantic information only but not as fast as when somatosensory information is also available during the early stages of learning.

## Introduction

Speaking starts with a message which the speaker wants to communicate, followed by an activation of concepts. This process is called initiation. Subsequently concepts activate words which may be inflected and ordered within a sentence with respect to their grammatical and functional role. This process is called formulation and starts with the activation of lemmas in the mental lexicon that correspond to lexical concepts within the semantic network. In a following step, the lemma’s corresponding word-forms are activated (Dell et al., 1997; Levelt et al., 1999). The phonological representation then is processed syllable by syllable by activating, executing, and monitoring a sequence of syllables. This process is called articulation and is thought to involve the mental syllabary (Levelt et al., 1999; Cholin, 2008; Brendel et al., 2011) as well as lower level motor and sensory processing modules. While the mental syllabary (Levelt and Wheeldon, 1994; Levelt et al., 1999) is accessed during phonetic encoding as part of the phonetic production process and comprises phonetic motor units it is hypothesized in our framework that an action repository is neurally connected with the mental lexicon comprising phonological, motor, auditory as well as somatosensory representations of all frequent syllables of a language (Kröger et al., 2009, 2011a,b). It is hypothesized that a hypermodal representation of these items (cf. Feng et al., 2011; Lametti et al., 2012) is stored in the action repository in the form of a cortical neural map which indicates an ordering of syllables with respect to syllable structure as well as with respect to phonetic features of the consonants and vowels building up each syllable (phonetic feature map, see Kröger et al., 2009; Kröger and Cao, 2015). This model has been embodied as quantitative computer model leading to results that approximate observed behavior but it is unclear how realistic the model is because some of its assumptions (especially the one concerning feature maps) are still not verified on the basis of neurophysiological findings.

It is still an open question how the knowledge and skill repositories mentioned above, i.e., how a mental lexicon and an action repository emerge and gather speech and language knowledge during speech acquisition and how both knowledge repositories are related to each other in order to allow speech processing (i.e., production as well as perception). The interaction between a mental lexicon and an action repository can be modeled if the syllabification process following the activation of phonological forms within the mental lexicon leads to syllable activation at the level of the action repository. This interface between mental lexicon and action repository does not exist at the beginning of the speech acquisition process, i.e., it is not available directly after birth. Moreover it can be assumed that the emergence of a phonological representation even for syllables, i.e., the emergence of a language-specific speech sound representation, as well as later on the emergence of phonological awareness (Castles and Coltheart, 2004) results from learning in early phases of speech acquisition, especially within the babbling and imitation phase.

Thus many models of speech production either focus on lexical linguistic processes and end with a phonological representation (e.g., Dell et al., 1997; Levelt et al., 1999; Levelt and Indefrey, 2004) or focus on the phonetic details and thus start with a phonological description of an utterance and give a detailed sensorimotor description of the speech production process (Saltzman and Munhall, 1989; Guenther et al., 2006; Guenther and Vladusich, 2012; Civier et al., 2013). In our approach we assume a phonological word-level representation as part of the mental lexicon while it is the task of the syllabification process to map these lexical phonological representations on syllabic phonological representations which are assumed to be part of the action repository (Kröger et al., 2014).

If we assume that only a sparse phonological representation exists at the beginning of speech acquisition (cf. Best et al., 2016), the emergence of the action repository as well as of the mental lexicon has to start with a sparse organization at the beginning of the acquisition process. Therefore we developed an approach comprising a direct neural association between conceptual lexical and sensorimotor syllabic representations of speech items. This approach elucidates how phonetic-phonological features and later on how a phonological representation of the target language emerges (Kröger and Cao, 2015). While the simulation of early phases of speech acquisition using this model was based on auditory stimuli in earlier simulations (ibid.) we now augmented the model in order to be capable of incorporating motor and somatosensory information.

It is the main goal of this study to evaluate how important the adding of somatosensory information is in order to learn phonetic-phonological features. For example the feature place of articulation is encoded in the acoustic speech signal in a very complex way and thus difficult for a listener to detect it from the acoustic speech signal alone. But place of articulation of consonants is easily detectable from somatosensory data like tactile feedback information from lips, tongue, and palate. Thus it can be assumed that somatosensory information plays an important role during those phases of speech acquisition coping with phonetic-phonological features like place of articulation.

## Materials and Methods

### Description of the Model

**FIGURE 1:**
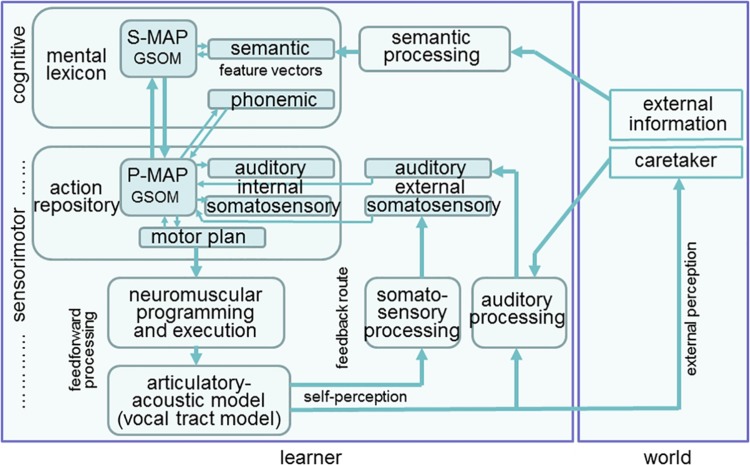
Structure of the model for simulating speech acquisition, speech production and speech perception.

The model is able to perform three working modes, i.e., learning, production, and perception. During learning, external knowledge – i.e., knowledge mainly gathered from interaction of the learner with its direct environment – is transferred to the learner (i.e., to the baby or toddler, also called “model”). This information is semantic information concerning words as well as auditory information generated by a caretaker. The neural model of the learner comprises a cognitive part and a sensorimotor part (Figure 1). The cognitive part consists of a growing self-organizing map (GSOM) representing words within a central neural map representing the mental lexicon. The growth process of that neural map takes place during learning. This neural map is also called semantic map or semantic feature map (S-MAP) because it is closely linked with the feature vectors representing each word, e.g., the word “mama” comprises semantic features like “is a human,” “is a female,” “is a part of parents,” etc. These semantic feature vectors are activated within the semantic state map, shown at the right side of the S-MAP in Figure 1. During learning words are ordered within the S-MAP with respect to the semantic features defining each word (Kröger and Cao, 2015). Neural representations of feature vectors can be activated at the level of the semantic state map and lead to an activation of a neuron, representing that word within the S-MAP, and vice versa.

The semantic state map together with the S-MAP and the phonemic state map form the mental lexicon. The phonemic state map comprises phonemic representations of syllables and words and emerges during speech acquisition. Semantic and phonemic state maps are part of short term memory and their neural activation patterns change from word activation to activation of the next word and so on while the S-MAP is part of long term memory and its model neurons directly represent words (ibid.). In our approach the phonemic state map is not directly linked to the S-MAP because only early phases of speech acquisition are modeled here. A neural connection with the S-MAP is formed later if the phonological representation or phonological awareness is developed. This process follows the processes described in this modeling study.

The sensorimotor part comprises the action repository or speech action repository in the context of our neural model and a feedforward-feedback loop for realizing the articulatory execution of motor plans (motor actions) and later on for the self-perception of somatosensory and auditory information generated by the model. A second GSOM, called phonetic map or phonetic feature map (P-MAP) is the central map within this speech action repository. The growth process of this neural map, like the growth process of the S-MAP, takes place during learning. During that growth process of the P-MAP an ordering of syllables occurs within this P-MAP, which is based on the auditory, somatosensory, and motor information. This information is temporarily activated at the level of the motor state, auditory state and somatosensory state map for a syllable if the syllable is planned and executed. The state maps are part of the short term memory and neural activation within these maps changes from syllable to syllable during speech production. The P-MAP itself is part of long term memory and each model neuron within this neural map represents a frequent and learned syllable of the target language like each neuron within the S-MAP represents a frequent and learned word. The P-MAP can be interpreted as a hypermodal feature map because the ordering of syllables occurring in this map is based on auditory, somatosensory as well as on motor information.

After syllable activation at the P-MAP level the feedforward processing of syllabic motor plans results in articulatory movements of vocal tract model articulators (vocal tract model, see Birkholz and Kröger, 2006; Birkholz et al., 2007) and the articulatory-acoustic part of this model generates (i) an acoustic speech signal and (ii) somatosensory signals (tactile and proprioceptive signals) which are processed by the feedback processing pathway (self-perception in Figure 1). The neuromuscular programming and execution is modeled in our approach by introducing control variables for model articulators. The time course of these control variables can be interpreted as model articulator movement trajectories and these variables are directly generated and controlled by vocal tract actions (Kröger and Birkholz, 2007). The feedback processing of the acoustic and articulatory signals leads to auditory and somatosensory syllable representations which activate the external auditory and somatosensory state maps and which can be compared to the already learned internal auditory and somatosensory representations for that syllable, stored in the neural associations between internal state maps and P-MAP.

### Neural Representation of Auditory and Somatosensory States

**FIGURE 2:**
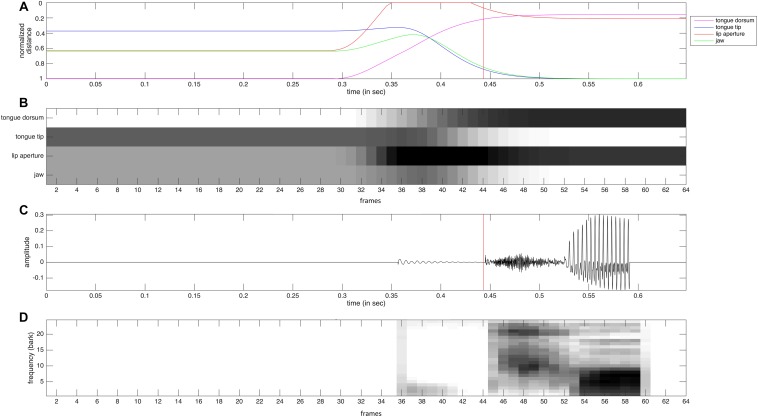
Somatosensory and auditory data **(A**,**C)** and the resulting neural representations **(B**,**D)** for a realization of the syllable [po]. **(A)** Normalized distance of tongue dorsum to palate (magenta) of tongue tip to alveolar ridge (blue), of lower to upper lips (red), and normalized height of jaw (green). **(B)** Neural representation of somatosensory data for four tiers (duration of each time frame is 10 ms). **(C)** oscillogram of acoustic speech signal. **(D)** bark-scaled neural spectrogram (duration of each time frame is 10 ms). The red vertical line in the data **(A**,**C)** indicates the beginning of noise burst after release of lip closure.

The auditory representation activated within the auditory state map can be interpreted as a neural version of a bark-scaled spectrogram (Figure 2D). This representation of a syllable is calculated from the acoustic signal (oscillogram, see Figure 2C). Each of the 24 rows of this two dimensional neural representation codes the acoustic energy within the frequency range of one bark region and each column represents a time interval of 10 ms (Cao et al., 2014). The degree of neural excitation within a frequency-time-slot is proportional to the acoustic energy within this slot. In the case of the syllable [po] displayed in Figure 2, a short and low level acoustic noise occurs at the beginning of lip closure at 0.35 s. A strong noise burst from 0.44 to 0.53 s appears after release of lip closure followed by a clearly visible vowel portion from 0.53 to 0.59 s with an initial formant transition, i.e., an initial increase in the frequency of F1 and F2 from 0.53 to 0.56 s.

The somatosensory data (Figure 2A) reflects the normalized distance between articulators (e.g., lower and upper lips) or between articulator and vocal tract wall (e.g., tongue tip with alveolar ridge or tongue dorsum with hard palate) for lips, tongue tip, and tongue dorsum. A value of zero reflects contact while a value of one reflects a far distance (e.g., wide mouth opening or low tongue position. In the case of the jaw the range between value one and value zero represents the range for low to high jaw position. The neural representation of these somatosensory data (Figure 2B) represents these distances. A small distance (i.e., articulator contact or high articulator position) is represented now by high neural activation (black), while a far distance is represented by low neural activation (white). Thus this neural information can be interpreted as somatosensory (i.e., tactile and proprioceptive), because it reflects articulatory contact as well as the positioning of articulators.

In the case of our sample syllable [po] we can clearly identify the time interval of labial closure from 0.35 to 0.43 s, an ascending movement of the tongue dorsum toward the [o]-target during this time interval, an ascending-descending movement of the jaw during this time interval in order to support the labial closure first and then to support the increasing oral front cavity for [o]. In addition we can clearly identify a descending movement of the tongue tip for the same reason, because the front part of the tongue must descend to effect the huge oral vocalic front cavity for [o] while the middle and back part of the tongue – i.e., the tongue dorsum – is involved in forming a vocalic constriction in the velar region of the vocal tract and thus increases in height.

### The Working Modes of the Model

The three working modes of the model are (i) learning during early phases of speech acquisition (babbling and imitation), (ii) production, and (iii) perception. In this paper we focus mainly on learning but learning needs the functionality of production as well as of perception. All working modes are currently limited in our model to the processing of monosyllables. That means that all words learned by the current model are monosyllabic.

#### Production

A concept of a word is represented by a model neuron within the S-MAP (Figure 1). This neuron is activated from a pattern of already activated semantic features at the semantic state map using a winner-takes-all procedure (Kohonen, 2001). Due to the S-MAP to P-MAP neural association this leads to the activation of a model neuron within the P-MAP and subsequently leads to an activation of a motor plan state followed by the generation of an articulation movement pattern and by the generation of an acoustic and articulatory speech signal (Figure 1). These acoustic and articulatory signals lead to an activation pattern at the level of the external auditory and somatosensory state maps via the self-perception feedback channels and the activation patterns of these external state maps can be compared with the internal auditory and somatosensory syllable representations which were activated from the P-MAP associations with the internal state maps (Figure 1) in order to guarantee a correct production of the syllable.

#### Perception

An auditory state representation is activated by an external speaker (e.g., caretaker, Figure 1) leading to a most activated winner-takes-all neuron at the P-MAP level. This results from the neural associations between external auditory state map and P-MAP (arrow from external auditory state map to P-MAP in Figure 1). Subsequently this leads to the activation of a winner-takes-all model neuron within the S-MAP via P-MAP-to-S-MAP association (arrow from P-MAP to S-MAP in Figure 1) and thus leads to the selection of a target concept at the level of the mental lexicon which then is activated in the semantic state map.

#### Learning

(i) Babbling starts with the activation of proto-vocalic, proto-CV and proto-CCV motor plans at the level of the motor plan state map within the action repository part of our model (Figure 1). “Proto-” means that these items are not language-specific but just raw or coarse realizations of vocalic, CV, and CCV syllables. If these articulatory movement patterns are executed via the feedforward and feedback route, neural activations occur not just within the motor state map but also in the external auditory state as well as in the external somatosensory state map. These three state representations or activations for each vocalic or syllabic item now form the input to the self-organizing phonetic feature map (P-MAP) for learning. Thus the phonetic feature map (P-MAP) is exposed to a set of sensorimotor learning items, i.e., to a set of syllables including motor states, auditory states as well as somatosensory states for each training item (Kröger et al., 2009). As a result, motor, auditory and somatosensory states are associated with each other for vowels and syllables. When this neural associative learning procedure is completed, auditory stimuli can be imitated because an auditory-to-motor state association has been learned now during babbling. Thus, the model can now generate an initial motor state if an auditory state is given.

(ii) Imitation starts with an auditory input generated externally (e.g., from a caretaker during learner-caretaker interaction, Figure 1). This auditory input, e.g., the word “ball,” leads to the activation of a winner-takes-all neuron at the P-MAP level. In parallel a winner-takes-all model neuron is activated at the S-MAP level on the basis of the same learner-caretaker interaction which is directed for example to the visible object “ball” via activation of the semantic feature vector of “ball” within the semantic state map (Figure 1). These parallel activations at S-MAP and P-MAP level simulate a learning situation, where a child (the learner) may draw his/her attention as well as the attention of the caretaker to an object (e.g., a ball which can be seen by both communication partners) and where the child now forces the caretaker to produce that word “ball,” i.e., to produce an auditory stimulus in parallel to the semantic network stimulation. Thus the concept “ball” is activated at the level of the semantic state network within the mental lexicon and the auditory representation of the same word is activated at the level of the external auditory state network within the action repository (Figure 1).

The resulting imitation learning within this word perception and word production scenario is a complex two stage process. Because each state activation (semantic as well as auditory level) leads to an activation pattern within the appropriate self-organizing map (S-MAP or P-MAP), neural associations are adapted between the semantic state map and the S-MAP at the level of the mental lexicon as well as between the auditory, somatosensory or motor state map, and the P-MAP at the level of the action repository. This leads to a modification of the ordering of syllables within the P-MAP. In the case of the mental lexicon this first stage process leads to an ordering of concepts within the S-MAP with respect to different semantic categories (cf. Kröger and Cao, 2015).

**FIGURE 3:**
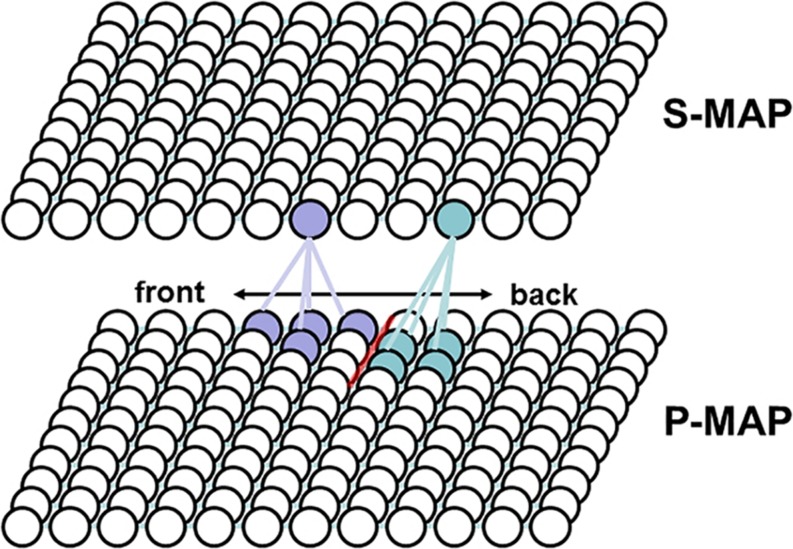
Example for two phoneme regions (light blue and light violet bullets, representing neurons or nodes) and one phoneme boundary (red line) at the P-MAP level. Light blue and light violet regions indicate the neurons or nodes representing phonetic realizations of two different concepts within the P-MAP and in addition neurons representing the concepts itself within the S-MAP. Lines between neurons of S-MAM and P-MAP indicate examples for strong associations between neurons or nodes.

The second stage of the imitation learning process leads to an association between S-MAP and P-MAP nodes which results from the temporally co-occurring S-MAP and P-MAP activation resulting from learning scenarios as exemplified above for the word “ball.” Later on during speech production the activation of an S-MAP node leads to an activation of a P-MAP node and vice versa in the case of speech perception (see Figure 3). Or in other words, imitation training leads to an association of phonetic forms (in the case of this study: V, CV, or CCV syllables) with meaning (in the case of this study: monosyllabic words). Due to the changes occurring within S-MAP and P-MAP as a result of the first stage of the imitation learning process a further adaptation or modification occurs for the neural associations between S-MAP and P-MAP in order not to change the already established correct associations between semantic and phonological forms (Cao et al., 2014 and see Appendix A in this paper).

As a result of imitation learning a bidirectional S-MAP to P-MAP association is established and it can be clearly seen, via this association, whether two syllables are phonetic representations of the same word or of different words. This implicates that an occurring phonetic difference within two syllables can be interpreted as a phonological contrast if the associated words are (i.e., if the meaning of the two syllables is) different. Rare cases like words conveying two meanings (e.g., “bank” of a river or “bank” as a financial institution) are not modeled in our approach because our approach is tested on the basis of a very limited model language. But because it can be assumed that the child learns one of the two word meanings first, while it learns the second meaning later, such rare cases lead to no complications from the phonological viewpoint of separating phonetic differences, because during the early learning process of phonetic separation of words only one word meaning is activated.

It has been shown by Kröger and Cao (2015) and it will be shown in this study that syllables are ordered with respect to phonetic similarity at the P-MAP level which is a typical feature of neural self-organization (Cao et al., 2014). Therefore neighboring syllables within the P-MAP in many cases only differ with respect to one segment and for this segment often only with respect to one phonetic-phonological feature. Thus within the P-MAP space we define the space occurring between syllables representing different meanings together with differences in specific segmental features of one segment as “phoneme boundaries” which is used here as an abbreviation for “boundary indicating a difference of at least one distinctive feature.”

As an example, at the level of the P-MAP syllables may be ordered with respect to phonetic features like vowel quality, i.e., vocalic phonetic features like high-low and front-back (Kröger and Cao, 2015). Thus a direction within the P-MAP may reflect the phonetic feature transition from high to low or from front to back vowels because a phoneme boundary concerning this feature occurs here (see Figure 3). It should be stated here that at the current state of the model the associations between S-MAP and P-MAP nodes define the word to syllable relation. This association does not affect the ordering of syllable items at P-MAP level (at phonetic level). All implicit syllable representations occurring within one “word region” at the level of the P-MAP, i.e., all syllable representations within the P-MAP representing one concept at S-MAP level, can be interpreted as phonetic realizations of syllables belonging to the same phonemic representation (see light blue and light violet regions in P-MAP in Figure 3). Thus, within the P-MAP we can find an ordering of phonetic syllable relations. Moreover we can find here boundaries for the separation of syllable realizations conveying different meanings. From this ordering and from the appearance of boundaries together with an already existing (intuitive) knowledge concerning syllable structure – including subsyllabic constituents like consonants and vowels – it is possible to extract phonological knowledge like “two neighboring P-MAP items conveying different meanings just differ in the first consonant of the syllable onset” or “this first consonant differs only in place or manner of articulation” or “two neighboring P-MAP items mapped conveying different meanings just differ in the vowel” and so on. This knowledge provides the basis to learn the phoneme repertoire, language-specific syllable structure rules, and the overall set of consonantal and vocalic distinctive features of the target language. In future versions of our model this knowledge will be saved within the phonemic state map (Figure 1). Thus the phonemic state map contains all target language phonological representations on syllable and segment level while the P-MAP only displays an ordering of phonetic realizations with respect to phonetic similarity from which phonological distinctions can be uncovered.

### Training Stimuli

The set of training stimuli consists of three realizations of 70 syllables, spoken by a 26 year old female speaker of Standard German (Cao et al., 2014; Kröger and Cao, 2015). These 70 syllables included five V-syllables (/i/, /e/, /a/, /o/, /u/), 5×9 CV-syllables combining each vowel with nine different consonants (/b/, /d/, /g/, /p/, /t/, /k/, /m/, /n/, and /l/) and 5 × 4 CCV-syllables combining each vowel with four initial consonant clusters (CC = /bl/, /gl/, /pl/, and /kl/). Thus, these 70 syllables (e.g., /na/) form a symmetrical shaped subset of syllables occurring in Standard German. This corpus was labeled as “model language,” because each syllable was associated with a word (e.g., {na}), i.e., with a set of semantic features (Kröger and Cao, 2015). The total number of semantic features was 361 in case of these 70 different words. The semantic processing for semantic feature selection for each word was done manually by two native speakers of Standard German (for details see Appendix Table A2 in Kröger and Cao, 2015). The chosen 70 words were the most frequent words occurring in a children’s word data base (Kröger et al., 2011a).

**FIGURE 4:**
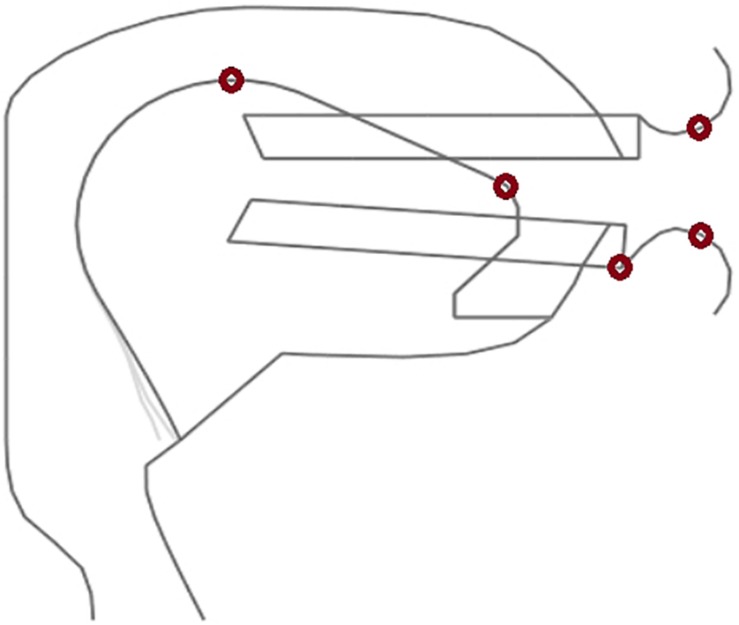
Midsagittal view of our vocal tract model showing the 5 tracking points (red circles) for calculating model articulator movement information. The point representing the jaw is attached to the front part of the lower teeth.

Each of the three acoustic realizations per syllable (word) was resynthesized using the procedure described by Bauer et al. (2009). The articulatory resynthesis procedure allowed a detailed fitting of the timing given in the acoustic signal to articulator movement on- and offsets as well as to sound target on- and offset (e.g., begin and end of closure in case of a plosive or nasal). Thus the articulatory resynthesis copied acoustic timing errors to articulation. Places of articulation, i.e., articulatory target positions were adapted with respect to the acoustic signal by manual fitting. In the cases of the acoustic stimuli used here places of articulation were always pronounced correctly by the speaker and thus the standard places of articulation as defined in the articulatory model for Standard German were used. This leads to a stimulus set of 210 items, each comprising a natural and a synthetic acoustic realization and a motor plan representation, stemming from the resynthesis process. The somatosensory representation was calculated from the movements of the model articulators of the vocal tract model during for each of the 210 resynthesized syllable realizations. Two lip points, two tongue points and one point of the jaw were selected and tracked within the midsagittal plane of the vocal tract (Figure 4). These points were tracked during execution of the resynthesized syllable items in order to get the articulator point trajectory information (cf. Figure 2A) from which the neural somatosensory state representation can be calculated for each of the 210 items.

### Training Procedure

An initial training cycle (training cycle 0) is executed in order to establish the initial GSOMs at the lexical and at the action repository level, i.e., the S-MAP and the P-MAP as well as to do an initial adjustment for the link weights of the bidirectional neural mapping (associative interconnection) between S-MAP and P-MAP (Cao et al., 2014). This training cycle is labeled as training cycle 0. Subsequently, fifty further training cycles were executed. Within the first 10 training cycles a *GSOM adaptation training* for both maps (P-MAP and S-MAP) is followed by an *interconnection adaptation training* for adjusting the associative interconnection network between both GSOMs and is followed by a *GSOM checking processes* which is executed during each training step (see Appendix Table A1). This training phase can be labeled as *babbling phase* because the P-MAP and S-MAP are trained here in isolation and only a very preliminary first associative interconnection network arises. Within the further 40 training cycles in addition an *interconnection checking process* is performed at the end of each training cycle which helps to establish an associative interconnection network between both GSOM’s. This training phase can be labeled as *imitation phase*. Within each training cycle each of the 210 items is activated 7 times (Cao et al., 2014), leading to 1470 training steps and thus 1470 adjustments of each link weight per training cycle. Beside the GSOM adaptation trainings and the interconnection adaptation trainings mentioned above additional GSOM adaptation trainings as well as additional interconnection adaptation trainings occur if this is demanded by the interconnection checking process done at the end of each training cycle. Thus a lower level GSOM checking process occurs after each training step and a higher level interconnection checking process occurs after each training cycle beginning with training cycle 11 (for details see Appendix Table A1).

In total twenty trainings with 50 training cycles each were simulated in order to end up with 30 instances of the trained model. Ten trainings were done using auditory information only, ten trainings were done using somatosensory information only and ten trainings used auditory and somatosensory information as input information for the self-organization of the P-MAP. Auditory information was taken from the natural items while the somatosensory information was taken from the resynthesized items, because no natural somatosensory data were available. Thus “auditory only trainings” and “auditory plus somatosensory trainings” can be separated in our study. Auditory trainings can be interpreted as purely passive trainings only using semantic plus auditory information while auditory plus somatosensory trainings in addition use information which stems from active articulation of the model during imitation. These later active trainings use information gathered from the resynthesized vocal tract movements (imitation movements).

## Results

### Evaluation of Number of Clear, Unclear, and Occupied Nodes at P-MAP Level

**FIGURE 5:**
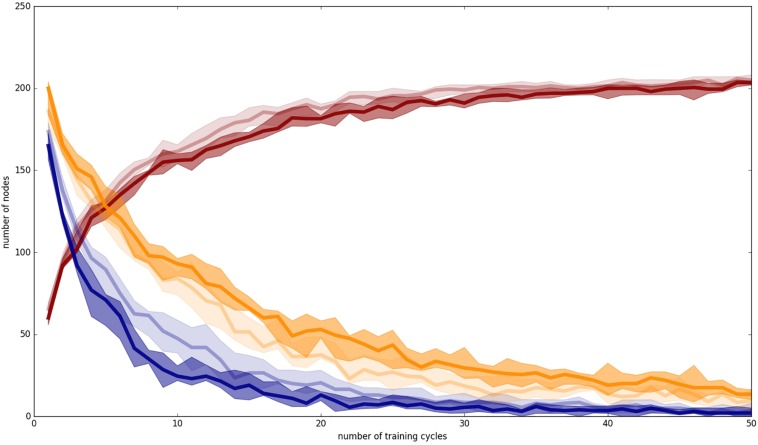
Number of unclear nodes (blue lines), clear nodes with non-separated items (yellow lines) and total occupied nodes (red lines) within the P-MAP for auditory plus somatosensory training (dark lines) and auditory only training (light lines); Median (50% percentile): thick line added by a shadowing between 2nd and 9th deciles, including 80% of the measured values (i.e., 10 and 90% percentiles).

In order to evaluate the increase in correct performance of speech perception and speech production as a function of increase in training cycles, three measures were taken, (i) the number of unclear nodes at P-MAP level (blue lines in Figure 5), (ii) the number of clear nodes with non-separated training items at P-MAP level (yellow lines in Figure 5), and (iii) the number of occupied nodes at P-MAP level (red lines in Figure 5). The terms “unclear node,” “clear nodes with non-separated training items” and “occupied nodes” are defined below in this section.

An unclear node at P-MAP level (blue lines in Figure 5) is a node which represents at least two training items belonging to two different syllables or words. Thus, an unclear node may lead to a failure in speech processing (perception or production) for these words, because they may be confused in speech perception as well as in speech production. In the case of more than 25 training cycles we found that the number N of unclear nodes leads to about 2^*^N different words which may be confused in production or perception, because after this number of training cycles the network is already differentiated and any unclear nodes do not represent more than two syllables or words.

In the case of auditory plus somatosensory training we get a mean value of *N* = 5 after 50 training cycles (Figure 5, dark lines), leading to a maximum of 10 of 70 words which could be confused in production or perception. In the case of auditory only training (Figure 5, light lines) we get *N* = 7, leading to 14 syllables or words which potentially could be confused in production or perception after 50 training cycles.

A clear node exhibiting non-separated training items at P-MAP level (yellow lines in Figure 5) is a node that represents at least two training items, but two training items which belong to the same syllable or word. In self-organizing networks it is desired that a node at P-MAP level represents a set of similar (phonetic) realizations of a syllable or word. This is called “generalization” and means that the network does not learn specific idiosyncratic differences of items representing one category (here: idiosyncratic differences of the phonetic realizations of a word) but generalizes toward the important (phonetic) features of and item in order to be able to differentiate items representing different words. Thus, the inverse of this measure (clear nodes representing more than one realization of the same syllable or word) represents the degree of overlearning. We can see that the number of this kind of nodes is low and thus the degree of overlearning is high, which may result from the fact that we train only three phonetic items per syllable, or word and thus are capable of learning specific features of each item because of the small number of training items per word. Thus, both of these facts, i.e., low number of items and close together grouping of items at P-MAP level, justifies the overlearning occurring in our simulations.

**FIGURE 6:**
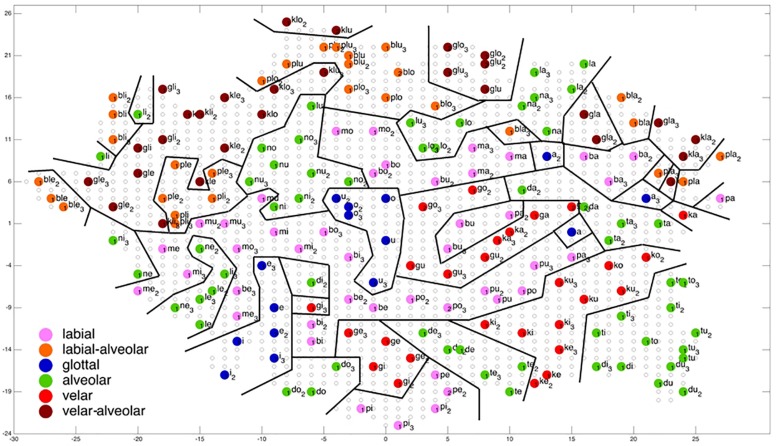
Display of feature regions for the consonantal feature place of articulation for auditory only training after training cycle 50 (training 2 of 10 trainings).

**FIGURE 7:**
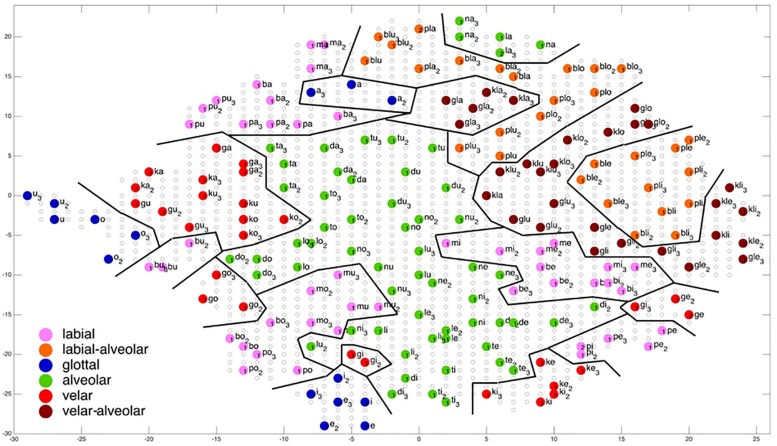
Display of feature regions for the consonantal feature place of articulation for auditory plus somatosensory training after training cycle 50 (training 2 of 10 trainings).

**FIGURE 8:**
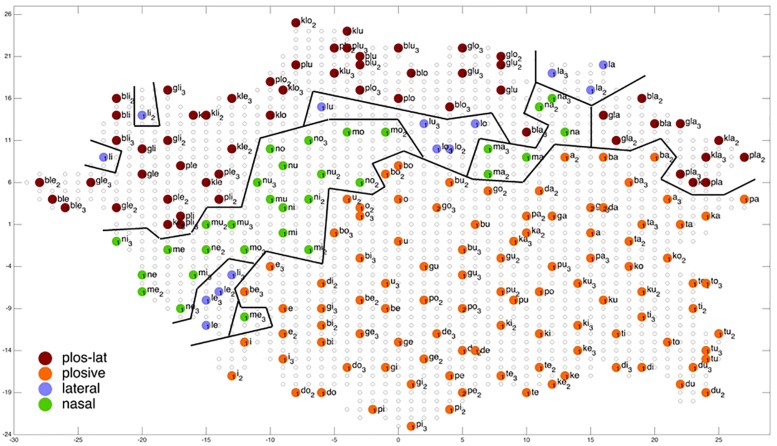
Display of feature regions for the consonantal manner of articulation for auditory only training after training cycle 50 (training 2 of 10 trainings).

**FIGURE 9:**
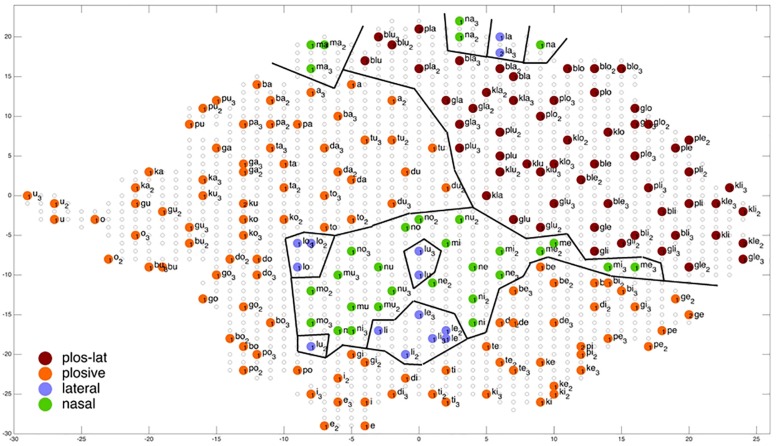
Display of feature regions for the consonantal feature manner of articulation for auditory plus somatosensory training after training cycle 50 (training 2 of 10 trainings).

But – as can be seen from Figures 6–9 – in most cases the nodes representing the same syllable or word are grouped closely together within the two-dimensional P-MAP. That means that learning leads to clear phoneme regions. These phoneme regions are not shown in Figures 6–9 because these phoneme regions in each case include 3 P-MAP nodes in maximum. The phoneme boundaries shown in Figures 6–9 are boundaries defined with respect to a specific phonetic-phonological feature contrast (distinctive feature contrast) and thus include more than one syllable or word. In the following they will be called “feature regions.”

In the case of auditory plus somatosensory training the degree of overlearning is lower in comparison to auditory only training (higher number of clear nodes with non-separated training items in the case of auditory plus somatosensory training: 20 nodes vs. 15 nodes in case of auditory plus somatosensory vs. auditory only training at training cycle 50). This indicates that the diversity of auditory only items is higher than of items including auditory and somatosensory information. This may result from the fact that somatosensory information is more useful for separating different places of articulation than auditory information. The use of somatosensory plus auditory information for example clearly separates different places of articulation with respect to labial, apical, and dorsal.

The number of occupied nodes at P-MAP level (red lines in Figure 5) is the sum of all nodes representing one or more training items (i.e., syllables). This number should be near the total number of training items if all training items are sufficiently learned and if in addition overlearning is strong and if in addition only few P-MAP nodes are unclear nodes. This is the case for both training modes. The number of occupied nodes is about 205 in the case of the auditory only training mode and about 203 in the case of auditory and somatosensory training mode after 50 training cycles. The lower number of occupied nodes in the second case may reflect the fact of a lower degree of overlearning in the case of auditory plus somatosensory training. This effect is significant (Wilcoxon rank sum text, two sided, *p* < 0.05) for most training cycles (see Appendix B).

Beside the results at end of training (training cycle 50) which we already stated above, it can be seen from Figure 5 that training leads to a faster decrease in number of unclear nodes in the case of auditory plus somatosensory training in comparison to auditory only training. A significant lower number of unclear nodes in the case of auditory plus somatosensory training compared with the case auditory only training is found for most training cycles (Wilcoxon rank sum text, two sided, *p* < 0.05 and see Appendix B). During later training cycles the number of unclear nodes further decreases but this difference is not anymore significant above training cycle 45 (Wilcoxon rank sum text, two sided, *p* > 0.05 and see Appendix B).

In the case of clear nodes representing more than one item of the same syllable (i.e., inverse degree of overlearning, yellow lines) it can be seen that overlearning increases significantly faster as well in the case of auditory plus somatosensory training in comparison to auditory only training (Wilcoxon rank sum text, two sided, *p* < 0.05 and see Appendix B).

### Evaluation of Ordering of Syllables at P-MAP Level

Figures 6–9 give a visual depiction how training items are grouped and ordered by neural self-organization within the P-MAP. Nodes of the P-MAP representing training items are marked by colored dots within the P-MAP while P-MAP nodes which do not represent a training item are indicated by light gray circles. The form and size of the map results from the training process as is described in Cao et al. (2014). If new items need to be represented in the map new nodes are generated and included in the map thus increasing its size. New nodes are always added at the edge of the map. Thus, the map’s form results from the addition of these nodes. The colors in Figures 6–9 represent different phonetic feature values with respect to place of articulation (labial to velar, see Figures 6, 7) and manner or articulation (plosive, nasal, lateral for CV-syllables, and plosive-lateral for the CCV syllables, see Figures 8, 9). The black lines indicate the boundaries of feature regions. It can be seen that the ordering with respect to place of articulation is better in the case of auditory plus somatosensory training (Figure 7) in comparison to auditory only training (Figure 6) after training is completed (training cycle 50), because the number of feature regions, i.e. the number of regions within the P-MAP with same “value” for a specific distinctive feature (regions edged by the black lines) is lower in the case auditory plus somatosensory training in comparison to auditory only training. No such clear difference occurs for manner of articulation (Figures 8, 9).

A further important result which can be directly deduced from a visual inspection of Figures 6–9 is that training items are grouped together for any given syllable. Thus, the three training items representing three realizations of one syllable or word are grouped together within the two-dimensional plane of the P-MAP. See for example the green dots in the upper right region of Figure 6 for the syllable or word {la} [-> (la1), (la2), (la3)] or the green dots indicating three representations of the syllable or word {na} [-> (na1), (na2), (na3)]. If a realization is missing in a figure, this realization overlaps with another realization of the same syllable or of another syllable.

This spatial grouping together of items of the same syllable or word within the space of the P-MAP indicates that different realizations of the same syllable or word are less different with respect to phonetic detail than realizations of different syllables. Moreover this result explains why overlearning can take place in our corpus and learning scenario: The P-MAP has enough nodes to represent each training item, but nevertheless a kind of generalization occurs because realizations of same syllables are grouped closely together.

Coming back to the display of feature regions, a further main result of this study is that the ordering of items with respect to place of articulation increases in case of auditory plus somatosensory training in comparison to auditory training, while no clear result can be drawn by comparing the feature regions for manner of articulation for both training modes. This is illustrated in Figures 6–9 which indicate that the number of feature regions within the P-MAP is higher in case of auditory only training (Figure 6) vs. auditory plus somatosensory training (Figure 7) for place of articulation.

The number of feature regions is lower for the consonantal feature manner of articulation (Figure 8) in comparison to the consonantal feature place of articulation (Figure 6) in the case of auditory training only (see also Kröger and Cao, 2015). If we compare the number of feature regions for manner of articulation for auditory plus somatosensory training (Figure 9) vs. auditory only training (Figure 8), it can be seen that the number of regions does not differ significantly. Thus the addition of somatosensory information to auditory information helps to separate place of articulation but not to separate syllables with respect to manner of articulation at the P-MAP level.

The faster learning (faster decrease in not clearly separated syllables) in case of auditory plus somatosensory learning can be seen by analyzing not just the phonetic feature separation at the P-MAP level after training cycle 50 (as done above: Figures 6–9) but by analyzing as well this feature separation at earlier training stages. This can be done by counting the number of feature regions for place and manner of articulation after 10 and 20 training cycles in comparison to 50 training cycles (Table 1) at P-MAP level. Figures 6–9 illustrate the term “number of feature regions”. Here we can find 39 feature regions in Figure 6, 19 feature regions in Figure 7, 11 feature regions in Figure 8 and 11 feature regions in Figure 9.

**TABLE 1:** Number of feature regions (mean value and standard deviation) for manner and place of articulation as function of number of training cycles (10, 20, and 50) for auditory only training (a) and for auditory plus somatosensory training (a+s).

**Training cycle**	**Manner (a+s)**	**Manner (a)**	**Place (a+s)**	**Place (a)**
10	9.8 ± 1.8	14.1 ± 2.4	22.4 ± 3.3	35.9 ± 3.5
20	9.1 ± 1.6	13.6 ± 2.1	22.1 ± 2.5	38.6 ± 3.4
50	10.8 ± 1.5	13.8 ± 2.3	22.0 ± 3.5	39.0 ± 3.6

*Each training mode has been executed 10 times (i.e., 10 trainings per training mode).*

Table 1 clearly indicates that already at training step 10 the number of feature regions is significantly lower for place of articulation in case of auditory plus somatosensory training (Wilcoxon rank sum text, two sided, *p* < 0.001) in comparison to auditory only training, while no such effect is found for the feature manner of articulation (Wilcoxon rank sum text, two sided, *p* > 0.05 except for training cycle 50, here *p* = 0.011).

## Discussion

This study illustrates how the emergence of an action repository can be modeled in a neural large scale model. Two training modes were chosen here, i.e., the “auditory only” and the “auditory and somatosensory” training mode. In the first mode the model is trained by auditory and semantic data while in the second case somatosensory information is added to the auditory information. This somatosensory information stems from the reproduction of syllables by the learner, i.e., by the model itself. From an earlier study using the same training set (Kröger and Cao, 2015) but focusing on auditory only training we know that in the case of this training set including V, CV, and CCV syllables the main feature for ordering syllables within a neural phonetic map is syllable structure (V, CV, and CCV), subsequently followed by the vocalic features high-low and front-back, followed by the feature voiced-voiceless for the initial consonant and then followed by the features manner and place of articulation for the initial consonant or consonant cluster.

In this study we focused our interest on the question of how learning of the features manner and place of articulation can be improved. It can be hypothesized that syllables may be ordered and thus learned more successfully if the feature place of articulation is learned as early and as fast as the feature manner of articulation. In the acoustic only training mode the feature place of articulation is learned later. In that case the ordering of the neural self-organizing map is better for manner than for place of articulation (Kröger and Cao, 2015). It can be hypothesized that place of articulation is perhaps learned earlier and as fast as manner of articulation if training not uses only auditory information but somatosensory information as well. This hypothesis is in line with the Articulatory Organ Hypothesis (Tyler et al., 2014; Best et al., 2016) which stresses the importance of the role of active articulators in production also for perception and thus for speech learning already in the first year of lifetime. Indeed an earlier and faster separation of syllables with respect to place of articulation and thus an earlier and faster learning of this feature has been found in this study for the case of availability of auditory and somatosensory information compared to the case of auditory information only. Because the feature place of articulation emerges later in training based on auditory information only (ibid.) the result of this current study indicates that somatosensory information, i.e., information based on articulatory imitation of syllables, helps to identify and to learn this important feature place of articulation already in early phases of speech acquisition.

Moreover it should be stated that at the end of training a correct performance of speech production and perception resulting from a correct and functionally ordered P-MAP is established as well in the case of auditory only training. Thus it can be hypothesized that somatosensory information may help to clarify which information within the acoustic signal is important in coding place of articulation, and may help to establish the feature place of articulation early in speech acquisition, but a correct performing speech processing model is established as well in the case of auditory only training. This result reflects the fact that place of articulation is sufficiently encoded in the acoustic speech signal mainly by formant transitions (Öhman, 1966) but these transitions are not easy to decode so that somatosensory information is helpful to decode this place information more easily.

Looking at the structure of the phonetic maps (P-MAPs) trained in this study as well as in an earlier study (Kröger and Cao, 2015) it can be stated that syllables are ordered with respect to different phonetic dimensions (features) like high-low, front-back, voiced-voiceless as well as for manner and place of articulation. This finding from our simulation studies finds correspondents in natural data stemming from neuroimaging studies (Obleser et al., 2004, 2006; Shestakova et al., 2004; Obleser et al., 2010) as well as from recordings of cortical activity using high-density multielectrode arrays (Mesgarani et al., 2014). The results of these studies show that a spatial separation of activation in cortical regions exits for different groups of speech items if these groups represent different phonetic feature values.

It should be kept in mind that our model on the one hand does not reveal a detailed phonetic-phonological mapping at the segment level. The implicit phonological representation introduced here is based on the associations between P-MAP and S-MAP as well as on the ordering of items within the P-MAP. On the other hand the boundaries shown in Figures 7–9 clearly indicate that boundaries emerge not only between the 70 types of syllables learned in these model simulations but also for different consonantal features occurring in the onset consonant of CV. Moreover, phoneme boundaries can also be found for different vocalic features as well as for different syllable structures like CV vs. CCV. These types of phoneme boundaries are not under discussion in this paper but are already shown as results of model simulations for different vowels in V-, CV-, and CCV-syllables in Kröger and Cao (2015) as well as for different syllable structures like V vs. CV vs. CCV in Kröger et al. (2011b).

Finally it should be stated that our training is based on semantic and sensorimotor phonetic information (auditory and somatosensory information) only. No phonological information is given directly here. The sensorimotor information comprises auditory information as it is generated by the caretaker as well as auditory, motor and somatosensory information generated by the learner itself during the process of word imitation. Thus our simulation approach clearly demonstrates that the emergence of phonetic features results from the ordering of items at the level of the P-MAP and that the emergence of phonological contrast as well results from this ordering together with information about which syllable is associated with which meaning (or word) generated at the S-MAP level. This later information is also available at the P-MAP level if a correct neural association between P-MAP and S-MAP results from the learning.

Our model starts with a direct neural association between semantic (or conceptual) and phonetic representations. That is the S-MAP and P-MAP associative interconnection. Other models like the GODIVA model (Bohland et al., 2010) directly start with hypotheses concerning the phonological representation by assuming a phonological planning module. But like in our model Bohland et al. (2010) assume predefined sensorimotor programs or predefined motor plans in terms of our model which are activated after passing the phonological planning phase. In GODIVA a speech sound map is assumed to represent a repository of motor plans of frequently used syllables which is comparable with the information stored in our P-MAP and its neural connection with the motor plan map. Bohland et al. (2010) as well see the syllable as the key unit for speech motor output. Like our P-MAP the speech sound map in GODIVA (ibid.) forms an interface between phonological encoding system (phonological plan and choice cells, ibid.) and the phonetic-articulatory system. But our model does not include a phonological encoding system because at this preliminary state our model is still limited to the production of monosyllables. Moreover sensorimotor programs for frequent syllables can be selected from speech motor map in full (ibid., p. 1509), which is comparable to an activation of a P-MAP node, leading to an activation of a specific motor program within the motor plan state map in our approach.

The concrete GODIVA model describes the temporal succession of phonological planning and motor execution. This is beyond the scope of our approach which is a purely connectionist model. Time is not an explicit parameter in our model but time is implicitly part of our model because motor plans as well as auditory and somatosensory states contain the information concerning the temporal succession and temporal overlap of articulatory actions as well as temporal information concerning auditory changes within a whole syllable. Thus our model can be seen as kind of “pre-model” describing how the knowledge for the speech sound map postulated in Bohland et al. (2010) could be acquired.

The HSFC approach (Hickok, 2012) as well as the SLAM model (Walker and Hickok, 2016) like our approach assume a direct neural connection between lexical modules (lemma level) to a syllable-auditory as well as to a phoneme-somatosensory module. These lower level modules define a hierarchy from lemma via syllable (including auditory feedback) to subsyllabic units like phoneme realizations. It is assumed in this approach that auditory feedback mainly influences syllable units while somatosensory feedback mainly influences segmental units. Like the DIVA and GODIVA model the HSFC approach does not include speech acquisition and thus does not speculate on syllabic or on segmental repositories like we do at least for the syllable level by introducing our P-MAP.

In summary, our neural model and the training scenario introduced here illustrate how a phonetic contrast can become a distinctive and thus phonological contrast during an extended training scenario if a semantic-phonetic stimulus training set is used covering the whole range of phonetic-phonological contrasts occurring in the target language under acquisition. The emergence of phonetic-phonological contrasts here results from the S-MAP to P-MAP association. But this knowledge now generated by learning needs to be generalized in order to develop the notion of different vocalic and consonantal distinctive features. This must be accompanied by already existing phonological knowledge concerning simple syllable structures (e.g., V, CV, and CVC,…) which already may exist at the beginning of babbling and imitation training. Thus, the central vehicle for locating this phonetic-phonological feature information is the neural P-MAP in our current model which forms a part of the action repository as well as the neural association occurring between P-MAP and S-MAP, but this information needs to be generalized and implemented in a phonological map which is not part of our current neural model. This may lead to a restructuring of the complex neural association of semantic and phonetic network levels in order to integrate a phonological representation layer.

## Conclusion

In this paper it has been illustrated how a neural realization of the action repository could be shaped and implemented in a computer based approach, how this action repository concretely emerges during speech acquisition and how phonetic items are ordered within this realization of an action repository. We were able to show that the occurring ordering of syllables within this realization of the action repository using GSOMs is the basis for a mental representation of phonetic features and that – due to an association between the action repository and the mental lexicon in early states of speech acquisition – first phonetic item clusters emerge which help to unfold the phonological organization of a target language.

It has been shown that a sufficient learning result is reached on the basis of auditory only training. Thus, motor representations leading to a correct imitation of syllables need not necessarily to be a part of speech (perception) learning, but the inclusion of imitation and thus the inclusion of production of speech items (e.g., of syllables) may lead to a faster acquisition of important features like place of articulation (cp. Iverson, 2010) in comparison to a passive learning processed only based on listening. This result implicates why children with severe speech motor dysfunctions are capable of learning to perceive and understand words like normal developing children (Zuk et al., 2018 for the case of childhood apraxia of speech), while learning correct word production of course is delayed, or perhaps never completed due to the existing motor dysfunction.

It is now necessary to further develop this neural simulation model of speech processing (production and perception) and speech learning in order to investigate the acquisition not just of a simple model language based on V-, CV-, and CCV-syllables and monosyllabic words but of a more complex real language. Furthermore it is important to extend the model with respect to the learning scenario. In our model, learning items are defined in advance but in reality the child actively shapes learning situations and thus actively shapes the set of training stimuli and especially the number of presentations and the point in time when the child wants to learn a specific word or syllable for example by turning the attention of the caretaker to a specific object within a communication situation. Thus, beside the caretaker also the child is able to actively control the learning process.

## Author Contributions

BK, MC, and TB programmed the software code. BK and TB conducted the experimental simulation. All authors designed the study, wrote, and corrected the manuscript.

## Conflict of Interest Statement

The authors declare that the research was conducted in the absence of any commercial or financial relationships that could be construed as a potential conflict of interest.

## Appendix A

*Learning algorithm for the neural network (two stage process)*. The whole neural network can be described as an interconnected growing self-organizing map (I-GSOM) which comprises two growing self-organizing maps (GSOM’s), i.e., a semantic map S-MAP and a phonetic map P-MAP where each node of one map is linked with each node of the other map and vice versa (associative bidirectional neural linking). The step-by-step update of all neural connection weights (link weights between nodes) during learning is described in detail in Cao et al. (2014). Both GSOM’s are trained and thus grow using the same neural learning and thus using the same neural principles for defining the link weights between GSOM and its associated state maps. The associated state map is the semantic state map in case of the S-MAP and is the auditory, somatosensory, and motor state map in case of the P-MAP (see Figure 1: semantic map beside S-MAP and internal auditory and somatosensory map beside P-MAP). Learning can be defined as a series of training steps. In each training step a word-syllable stimulus pair is applied to the state maps. First, the node within each GSOM is determined which is representing the stimulus best, i.e., which is most similar to the stimulus (winner node), and the link weights of this node and of nodes in a defined neighborhood of the winner node are modified in direction toward the stimulus, i.e., the link weights between state maps and GSOM are modified in a way, that the winner node now is more similar to the training stimuli than it was before. The degree of approximating the stimulus in one training step is defined by the learning rate of the neural model. This learning is called *GSOM adaptation training* and done independently for both GSOMs. It leads to a self-organization of both GSOMs: (i) The nodes representing words are ordered with respect to all semantic features within the S-MAP which are inherently included in the set of word training stimuli. (ii) Syllables are ordered with respect to all phonetic features within the P-MAP which are inherently included in the set of syllable training stimuli. The result of this learning is also called neural self-organization and the associated maps are called self-organizing feature maps (Kohonen, 1982, 2001, 2013).

In order to allow a growth of these maps during this learning process the original algorithm developed by Kohonen (ibid.) has been modified as described by Alahakoon et al. (2000). While the modification of link weights is similar in SOM’s and GSOM’s a growth criterion needs to be defined in the case of a GSOM. Therefore each training stimulus is matched with each node of the already existing GSOM and the error with the best matching neuron within the GSOM is accumulated over successive training steps until a threshold value is reached indicating that a new node needs to be added to the GSOM in order to allow a better matching of stimuli and GSOM neurons. This growth process occurs together with self-organization of each GSOM and is part of the GSOM adaptation training.

In the babbling phase an adaptation of the P-MAP only is done on the basis of syllable stimuli. During the imitation phase the S-MAP is adapted in parallel. For auditory only training the somatosensory training data are not applied and vice versa for somatosensory alone training no auditory training data are applied. In case of auditory plus somatosensory training the *whole* set of training data is applied. Because of the similarity of motor and somatosensory training data the training of the P-MAP is done by using auditory and/or somatosensory data only in case of this study.

In addition to the GSOM adaptation training the training or learning of the associative mapping between both GSOM’s, i.e., the development of the associative neural interconnections between both GSOM’s needs to be done. This training is called *interconnection adaptation training*. The link weights of a neural interconnection link are modified (i.e., increased) only if winner-takes-it-all nodes occur simultaneously in both GSOM’s for a given stimulus pair (i.e., a word-syllable pair). “Simultaneously” means that a combined word-syllable stimulus is applied to the I-GSOM leading to specific simultaneous activations of all nodes. The link weights between these two winner neurons are modified in a way that the interconnection between both winner neurons is strengthened in both directions between both GSOM’s. If no winner-take-all neuron occurs for a specific stimulus in one of the GSOM’s this GSOM is not able to identify a node as a good representation for a stimulus. In this case further GSOM adaptation training steps are needed. Whether those interim GSOM adaptation trainings are needed is checked by a *GSOM checking process*, which is executed in combination with each potential interconnection adaptation training step (see Appendix Table A1).

**TABLE A1:** Organization of the whole training of the I-GSOM neural network.

**Babbling training**	
• *P-MAP adaptation training* on basis of 5 training cycles for the syllable stimulus set (5 × 7 × 210 training steps randomized)	
**Imitation training**	
• *P-MAP and simultaneous S-MAP adaptation training* on the basis of 50 cycles for the word-syllable stimulus set (50 × 7 × 210 training steps, randomized)	
• At end of each adaptation step (i.e., 50 × 210 times in total): *GSOM checking process*	
	• If GSOM checking process is positive: *interconnection adaptation training*
	• If GSOM checking process is negative: *GSOM reinforcement and GSOM reviewing training* (adaptation of P-MAP and of S-MAP for N_*u*_ “unsolved” stimuli; *N*_*u*_ < 210)
• Beginning with cycle 11: at end of each training cycle (i.e., 40 times in total): *interconnection checking process*	
	• If interconnection checking process is positive: return to normal *P-MAP and simultaneous S-MAP adaptation training* (first two main black bullets of imitation training)
	• If interconnection checking process is negative: add an *interconnection link forgetting process* before returning to the *interconnection checking process*

*In each training cycle all 210 the stimuli are applied 7 times randomly ordered. GSOM adaptation training includes adaptation of link weights between a GSOM and its state maps as well as growth of the GSOM.*

The GSOM checking process identifies so-called “high-density nodes,” i.e., nodes which represent more than one stimulus within the P-MAP or within the S-MAP. In this case a modified GSOM adaption training will be inserted after the GSOM checking process. The modification is that during the GSOM adaptation training only those stimuli are applied to the neural network which are not resolved thus far. This modified GSOM adaptation process thus represents a process in which the learner is aware that there are still some words and syllables which cannot be produced correctly and thus are not perceived correctly by the caretaker. This modified GSOM adaptation training is called *GSOM reinforcement training* (see Appendix Table A1). The word “reinforcement” is chosen because it is assumed that the caretaker (as well as the child) is aware of this situation and thus concentrates on learning of “difficult” words and syllables. At the end of a GSOM reinforcement training phase a *GSOM reviewing training* phase is included which – like the normal GSOM adaptation training for each GSOM – again includes all 210 stimulus pairs i.e., recapitulates all items which were are ready learned and which are still to learn. This GSOM reviewing training is important to guarantee that the network does not “overlearn” the difficult words or syllables trained in a GSOM reinforcement training and thus forgets the other earlier learned words or syllables.

Moreover it may happen that a wrong link has been established within the associative neural interconnection network between both GSOMs. This may happen if a winner node is identified in one of the GSOMs for a specific word or syllable but this winner neuron later during learning turns to represent a different word or syllable. This may happen because the whole learning process is highly dynamic. Thus link weights are allowed to change with respect to learning rate and thus are quite flexible. In order to be able to cope with such situations a further higher level checking process, called *interconnection checking process* is included in the whole training procedure. This process starts if already 10 main training cycles have been executed in order to guarantee that a preliminary associative interconnection network is already grown between both GSOMs. Normal training is continued if the interconnection checking process allows it (see Appendix Table A1). Otherwise, the interconnection checking process demands a change in link weights of the identified wrong associative interconnections towards smaller values. This procedure is called *interconnection link forgetting process* (“link forgetting procedure” following Cao et al., 2014). This process needs to be introduced explicitly because associative learning as it is used within the interconnection adaptation training can only increase link weights. These interconnection checking processes are applied after each fully completed training cycle starting with training cycle 11 and thus occur 40 times in total in our learning scenario (Appendix Table A1).

## Appendix B

*Significance levels for difference of median values*. This appendix gives the significance levels for the difference of median values of dark vs. light lines in Figure 5, i.e., differences between the median values in case of auditory plus somatosensory training (Figure 5, dark lines) and the median values in case of auditory only training (Figure 5, light lines) for the three measures for nodes listed in Appendix Table B1. No correction of p-values was performed despite testing at each of 50 points in time representing different training cycles.

**TABLE B1:** Significance level for median values of three measures (i) the number of unclear nodes at P-MAP level (blue lines in Figure 5), (ii) the number of clear nodes with non-separated training items at P-MAP level (yellow lines in Figure 5), and (iii) the number of occupied nodes at P-MAP level (red lines in Figure 5) for the comparison of auditory plus somatosensory training (Figure 5, dark lines) with auditory only training (Figure 5, light lines) for each training cycle (1–50).

**Number of training cycle**	**Unclear nodes (blue lines)**	**Clear nodes (yellow lines)**	**Occupied nodes (red lines)**
1	^*^	^*^	^*^
2	^∗∗∗^	n.s.	n.s.
3	^∗∗^	^∗∗^	^∗∗^
4	^∗∗∗^	^∗∗∗^	n.s.
5	^∗∗^	^*^	^∗∗^
6	^*^	^*^	^∗∗^
7	^∗∗∗^	^∗∗^	^∗∗∗^
8	^∗∗∗^	^*^	^∗∗∗^
9	^∗∗∗^	^*^	n.s.
10	^∗∗∗^	^∗∗^	^∗∗^
11	^∗∗^	^*^	^*^
12	^∗∗∗^	^∗∗^	^∗∗^
13	^∗∗^	^∗∗^	^∗∗^
14	n.s.	^∗∗∗^	^*^
15	n.s.	^∗∗∗^	^∗∗∗^
16	^∗∗^	^∗∗^	^∗∗∗^
17	^∗∗^	^*^	^*^
18	^∗∗∗^	^∗∗^	^*^
19	^∗∗^	^*^	^*^
20	^∗∗∗^	^∗∗∗^	^∗∗∗^
21	^∗∗^	^∗∗^	^*^
22	^∗∗^	^∗∗∗^	^∗∗∗^
23	^∗∗^	^∗∗^	^∗∗^
24	^∗∗^	^∗∗^	^*^
25	n.s.	^∗∗^	^∗∗^
26	n.s.	^*^	^*^
27	^∗∗^	^∗∗^	^∗∗^
28	^*^	^∗∗∗^	^∗∗∗^
29	^*^	^∗∗∗^	^∗∗∗^
30	^*^	^∗∗^	^∗∗^
31	n.s.	^∗∗^	^∗∗∗^
32	^*^	^∗∗^	^*^
33	^*^	^∗∗^	^∗∗^
34	^∗∗^	^∗∗^	^∗∗^
35	^*^	^∗∗^	^∗∗^
36	n.s.	^*^	^∗∗^
37	^∗∗^	^∗∗^	^*^
38	^∗∗^	^*^	^*^
39	^*^	^∗∗^	^∗∗^
40	n.s.	^*^	n.s.
41	n.s.	^∗∗^	^*^
42	^*^	^∗∗^	^*^
43	n.s.	^*^	^∗∗^
44	^*^	^*^	^*^
45	^∗∗∗^	n.s.	n.s.
46	n.s.	n.s.	n.s.
47	n.s.	^∗∗∗^	^∗∗∗^
48	n.s.	^*^	^*^
49	n.s.	^*^	^*^
50	n.s.	n.s.	n.s.

*Significance levels: ^*^<0.05, ^∗∗^<0.01, and ^∗∗∗^<0.001; n.s., both median values are not significantly different.*
